# Diagnostic safety in the policy landscape - a comparative policy document analysis of Australian and Aotearoa New Zealand vs US health care and research policy

**DOI:** 10.1186/s12913-025-13782-7

**Published:** 2025-12-05

**Authors:** Maria R. Dahm, Rose M. Carey, Leslie Tucker, Rebecca Haddock, Mark L. Graber

**Affiliations:** 1 Institute for Communication in Health Care (ICH), College of Arts and Social Sciences, Australian National University, Canberra, ACT 2601 Australia; 2 Present Address: School of Medicine, Deakin University, Geelong Waurn Ponds Campus, Locked Bag 20000, Geelong, VIC 3220 Australia; 3 Present Address: Centre for Quality and Patient Safety Research, Institute for Health Transformation, Deakin University, Geelong, Australia; 4 ANU Medical School, Australian National University, Canberra, ACT 2601 Australia; 5 LTucker Consulting, Alexandria, VA USA; 6 Australian Healthcare and Hospitals Association, 8/2 Phipps Cl, Canberra, ACT 2600 Australia; 7 Community Improving Diagnosis in Medicine (CIDM), Plymouth, MA 02360 USA

**Keywords:** Diagnostic errors, Patient safety, Diagnostic safety, Document analysis, Health policy, Research policy, Emergency medicine

## Abstract

**Background:**

Diagnostic safety, a subset of patient safety, ensures safe, high-quality care in the diagnostic process e.g. through reporting and evaluating near-misses and errors. It involves healthcare policy (e.g. incident reporting guidelines) and research policy (e.g. research funding). To date, policy attention to diagnostic safety has been limited.

**Methods:**

Across United States (US) versus Australian and Aotearoa New Zealand (AUS/AoNZ) policy contexts, we systematically identified relevant policy documents from national health quality organisations and Emergency Medicine (EM) specialist colleges and compared the development and integration of diagnostic safety into policy. We adopted a directed policy document analytical approach (READ (Ready materials, Extract data, Analyse data, Distil findings) to develop comparative frameworks for diagnostic safety policy and embedded a case study on Emergency Medicine guidelines.

**Results:**

We identified 237 publicly available, written policy documents (AUS/AoNZ: *n* = 151; US: *n* = 86) and 36 EM guidelines AUS/AoNZ: *n* = 16; US: *n* = 20) from national US and AUS/AoNZ health quality organisations and EM specialist colleges. The majority of policy documents (55%) were published between 2019 and 2023. US policy documents had a greater dedicated diagnostic safety focus (*n* = 58, 67%) compared to a generic patient safety focus (*n* = 28; 33%) and had higher emphasis on research compared to diseases. AUS/AoNZ documents focused more on generic patient safety (*n* = 102, 68%) than diagnostic safety (*n* = 49, 32%) and concentrated more on diseases than research. The majority of EM guidelines (AUS/AoNZ: 81%, US: 75%) contained diagnostic safety comments, but overall 20% focused on treatment rather than diagnosis. US EM guidelines showed greater legal considerations.

**Conclusions:**

Awareness of diagnostic safety as reflected in healthcare and research policy is growing. Identifying country-specific differences can inform future strategic policy development and target areas that have received limited attention.

## Introduction

Diagnostic errors - untimely, inaccurate or ineffectively communicated explanations of patients’ health problems [1] – generally occur in 5–15% of cases [2, 3], and are among the top causes of serious iatrogenic patient harm including death [4]. Diagnostic safety, a field of patient safety concerned with safe care during the diagnostic process, includes attention on clinical reasoning [5], communication [6], clinical education [7], and reporting and evaluation of near-misses and diagnostic errors [8].

With the establishment of a national patient safety organisation and surveillance system in 1987, Australia showed early leadership in the patient safety movement [9]. In 2005, the United States similarly adopted a system of patient safety organisations and a national patient safety database [10]. At the time, neither country contemplated diagnostic error in its standards or guidance documents. It would take longer for this to change, starting with the publication of the National Academies of Science, Engineering and Medicine (NASEM) report “Improving Diagnosis in Health Care” [1]. That report, which had global reach, put a spotlight on the immense human and financial toll of diagnostic error and issued a clear call to clinical leaders and public policy makers that reducing harms from diagnostic error amounts to a moral, professional, and public health imperative.

Diagnostic safety has been slow to be adopted into clinical, public health and funding policy in the US [11] and internationally, including in Australia [12], in the decade since the NASEM report was released. Policy attention on diagnostic safety may be stalled due to a Catch-22 situation where the dedicated resources needed to build evidence and focus attention among clinicians, health organisations and policy makers have been limited due to a lack of: awareness [13, 14], reliable measurements [15], evidence-based interventions [11], ownership due to professional siloes [12] and dedicated research and funding [16].

A limited understanding of diagnostic safety in current policy can hinder our ability to effectively address this critical patient safety issue, preventing the development of targeted interventions and impeding progress in reducing diagnostic errors. Australia’s early leadership in patient safety and the US’ recent focus on diagnostic safety provide a valuable comparative lens for examining policy development in this critical area. In our exploration, we extend the Australian context to include Aotearoa New Zealand due to their longstanding history of joint specialist colleges overseeing medical and specialty training and accreditation, including e.g. surgery, general practice, and emergency medicine [17, 18].

Here, we conceptualise two broad policy areas related to diagnostic safety including: 1) healthcare policy, focused on e.g. diagnostic guidelines, practice and/or accreditation standards, incident reporting policies, digital health decision support, and safety and performance measures; and 2) research policy, focused on e.g. making diagnostic error a research priority and ensuring dedicated research funding.

This study aimed to 1) examine and compare whether and to what extent diagnostic safety is represented in United States (US) versus Australia and Aotearoa New Zealand (Aus/AoNZ) healthcare and research policy contexts (i.e. with what focus and level of consideration), and 2) to map diagnostic safety policy and practice in their wider national contexts by adopting a document analysis approach with a case study focus on emergency medicine. While diagnostic safety is paramount across all health care settings [1], emergency medicine was chosen for the case study due to its inherent time-sensitive environment where diagnostic accuracy is critical and errors can have immediate, severe consequences [3]. By comparing these healthcare systems, this study aims to identify key factors influencing diagnostic safety policy development and implementation, with the ultimate aim of providing a strong foundation to guide future national policy efforts.

## Methods and analysis

### Design and data collection

This policy document analysis is a part of larger multi-national project focused on analysing diagnostic safety in policy and emergency care practice [19]. Given the broad scope of health care and research policy, this study focused on overall national (instead of state-based) healthcare policy, acknowledging differences in population size and state-based health system complexity and fragmentation between United States, Australia and Aotearoa New Zealand, and ensuring proportionality of results by aligning the data collection scope with the scale of the different countries. In addition to diagnostic safety in general, this study also concentrated on emergency medicine as one subspecialty subject to higher rates of diagnostic errors compared to other health settings [3].

We adopted a policy document analysis approach with an embedded case study focused on emergency medicine guidelines. To ensures a rigorous and transparent methodology for our comparative policy document analysis, MRD and RMC followed the systematic READ approach (*Ready materials, Extract data, Analyse data, Distil findings*) [20]. Here, we broadly conceptualised “policy documents” as publicly available, written documents such as reports, websites, guidelines related to healthcare or research policy produced by national health quality, policy and/or funding organisations. We focused on identifying policy documents and emergency medicine guidelines produced by national organisations across two health system contexts 1) United States and 2) Australia and Aotearoa New Zealand (AUS/AoNZ).

Compared to the United States, AUS/AoNZ have comparable universal public and private health systems with their own yet highly similar national organisations responsible for ensuring safety and quality of care as well as research funding. Crucially, AUS/AoNZ also share standards and specialist colleges that jointly oversee medical training and specialty training and accreditation across both nations [17, 18]. For example, AUS/AoNZ share a specialist Emergency Medicine college (Australasian College of Emergency Medicine (ACEM)) which produces joint policy and guidelines for both countries but also occasionally releases documents targeted at one country. Considering the substantial similarities in healthcare systems and shared governance of training and accreditation entities we combined documents from AUS/AoNZ for this analysis. However, it is important to note that this decision did not result in a ‘doubling’ of identified documents for AUS/AoNZ compared to the United States.

All sourced policy documents are publicly available and do not require ethical approval for secondary analysis. Unique document IDs and references are cited as illustrative examples in the findings section.

### *Ready* materials

To identify (*ready*) relevant policy documents, MRD and RMC comprehensively searched (without date restrictions) the main websites and resource libraries of national health quality organisations, and policy and funding agencies (e.g. National Health and Medical Research Council (NHMRC, Australia), Agency for Healthcare Research and Quality Health (AHRQ, US)) between September 2022 and March 2023. We excluded the following types of documents: literature reviews (including scoping review, environmental scans), academic papers, media releases/news story, and documents related exclusively to COVID-19. In our systematic approach, we briefly read [21] all relevant documents contained on the identified source sites (including documents referenced therein) and searched for the presence of keywords: diagn*, error, *certain and fail*; then followed the systematic procedure outlined below in the *Initial* *Extraction* section. The keywords were chosen to allow us to capture core aspects related to diagnostic safety and the diagnostic process; the act of diagnosing symptoms or diseases, running diagnostic tests, establishing diagnostic criteria, and navigating diagnosis in (un)certain conditions, alongside common expressions of errors or failures within the diagnostic process. To avoid duplication, we cleaned data by removing individual chapters of larger reports that had appeared in resource libraries and were originally logged as separate documents. We retained the complete reports.

To identify (*ready*) emergency medicine guidelines, we sourced current and publicly available emergency medicine clinical guidelines from two emergency medicine colleges: 1) United States: all documents listed under the heading clinical policies on the website of the American College of Emergency Physicians (ACEP) [22]; and [2]) AUS/AoNZ all documents tagged as ‘Clinical Guideline’ in the resource library of ACEM [23].

## Analysis

### Initial extraction

Unless screening keywords were only present in the documents’ glossary, we saved documents with a unique ID in a local Excel spreadsheet. For all documents and emergency medicine guidelines, we *extracted* information on producing country, institution, year, document name and type, main topic (such as disclosure, transition of care), length and initial researcher impressions. Following this initial generic extraction, we used slightly different approaches for the *extraction* and *analysis* of policy documents and emergency medicine guidelines. Further extraction went hand in hand with developing analytical frameworks for documents and emergency medicine guidelines alike.

### Extract and analyse data – policy documents analytical framework

**Fig. 1:**
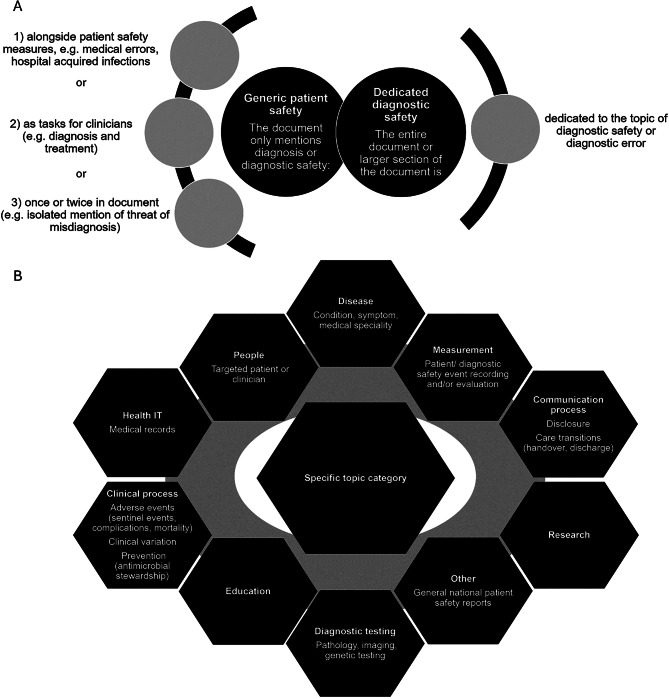
Policy analysis framework. (**A**) Specific focus and document features for each overarching category. (**B**) Specific topic categories across all documents

Given the length of identified policy documents [21], MRD and RMCs close reading, data extraction and analysis [20] was also guided by screening for keywords: diagn*, error, certain, and fail*. This approach assisted us in developing and applying a targeted deductive coding framework for generic patient vs dedicated diagnostic safety which aided in *extracting* further researcher impressions and representative quotes informing *analysis* of specific topic categories across all documents (see Fig. 1). This framework could be applied, and adapted to assess diagnostic safety in policy context in other countries.

While topics overlapped (especially in relation to disease, people, research and clinical process), we chose to code into only one exclusive category guided by first topic aspect mentioned in the title of the document. For example, a document entitled “Disclosure of harm following an adverse event” was coded for *communication* rather than *clinical process (adverse event)*. This exclusive coding allowed us to compare focus areas between the different contexts.

### Extract and analyse data – emergency medicine guidelines analytical framework

For emergency medicine guidelines, we immersed ourselves in the documents during close reading [20] without guidance from screening keywords; we read all sections including appendices. Following discussions, MRD and RMC again developed a coding framework to guide data *extraction* (including quotes) and *analysis* of: 1) descriptive categories (e.g. page numbers, target patient population, disease/focus, equivalent guidelines in the other region); 2) explicit comments related to diagnostic safety, the use of scientific evidence or legal consideration/limitations and 3) diagnostic focus. Diagnostic focus was assessed using a priority-based approach concentrating on mentions of symptom, suspected diagnosis, or (confirmed) diagnosis firstly in the guidelines’ inclusion criteria, or purpose/scope statement, and secondly in the guidelines’ title. We did not assign the higher priority to guideline titles as their brevity often omitted words like “suspected” whereas purpose statements made the focus on such potential diagnoses clear. We used titles where documents lacked other relevant statements.

Diagnostic focus categories and criteria location within the document are summarised in Table 1:

**Table 1:** Diagnostic focus categories and criteria location for Emergency Medicine guideline analysis

Diagnostic focus categories	Criteria location and example
Working towards diagnosis	• make a diagnosis (symptom and/or suspected diagnosis in purpose statement/title, e.g. “Title: Adult with a blunt head injury”)
Starting with diagnosis	• manage established diagnosis (diagnosis in purpose statement/title; e.g. “Inclusion Criteria. This guideline is intended for adult patients aged 18 years and older presenting to the ED with acute ischemic stroke.”)
Dual purpose	• make a diagnosis and manage it (symptom *and* diagnosis, or suspected diagnosis *and* diagnosis in purpose statement/title, e.g. “Inclusion Criteria. This guideline is intended for patients presenting to the ED with acute, nontraumatic abdominal pain and possible or suspected appendicitis.”)
Not diagnosis related	• (neither symptom, suspected diagnosis nor diagnosis in purpose statement/title, e.g. “Title: Guideline on procedural sedation”)

## Study results

The following sections and discussion, provide our interpretation of the findings (*distil*) [20] from the analysis of policy documents and emergency medicine guidelines.

### Policy documents

#### Overall findings across health system contexts

Across both health system contexts, we identified 237 policy documents (AUS/AoNZ: *n* = 151 (AUS: *n* = 110; AoNZ: *n* = 29, AUS&AoNZ: *n* = 12); US: *n* = 86), and 36 emergency medicine guidelines (AUS/AoNZ: *n* = 16; US: *n* = 20).

**Fig. 2:**
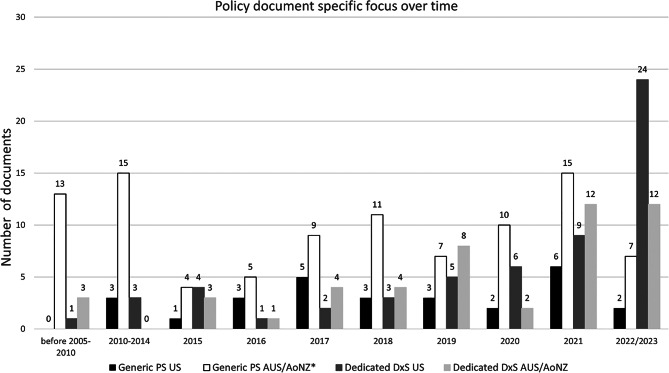
Specific policy document focus over time. PS: patient safety, DxS: diagnostic safety. *n = 6 documents were undated

Sourced policy documents were published between 2000 and 2023, with 16% (*n* = 38) published before 2015. Overall, more than half of all documents (*n* = 130, 55%) were published between 2019 and 2023, with 40% (*n* = 52) of all generic patient safety documents and 73% (*n* = 78; US: *n* = 44 (75%); AUS/AoNZ: *n* = 34 (69%) of dedicated diagnostic safety document published in this period. Only 7% (*n* = 7) of all documents with a diagnostic safety focus were published before 2015 - the year in which the milestone NASEM report [1] on improving diagnosis was published. As presented in the detailed breakdown in Fig. 2, the documents’ specific focus showed an emergent upward trajectory for a dedicated diagnostic safety focus as compared to the patient safety focus which remained relatively stable across the years.

Table 2 provides an overview of policy documents identified for each health system context including number of pages as well as the document’s specific focus category. Overall, across all contexts, a little more than half of documents (*n* = 130, 55%) focused on generic patient safety with the majority produced in AUS/AoNZ (*n* = 102, 78% (AUS: *n* = 69; AoNZ: *n* = 24, AUS&AoNZ: *n* = 9)) and less than a third in the US (*n* = 28, 22%). Across all documents, slightly less than half (*n* = 107, 45%) were dedicated to diagnostic safety with roughly equal distribution among both contexts (AUS/AoNZ: *n* = 49 (AUS: *n* = 41; AoNZ: *n* = 5, AUS&AoNZ: *n* = 3); US: *n* = 58).

**Table 2:** Policy documents across all health system contexts, pages and producing organisations, and specific focus category

	Health system context		
Document characteristics	United States	AUS/AoNZ	Total
Policy, documents, n (%)	86 (36%)	151 (64%)	**237 (100%)**
Number of pages, n (%)	2,635 (19%)	11,448 (81%)	**14,083 (100%)**
Number of organisations, n (%)	14 (54%)	12 (46%)	**26 (100%)**
Generic patient safety (PS), n (%)	28 (22%)	102 (78%)	**130 (55%)**
Dedicated diagnostic safety (DxS), n (%)	58 (54%)	49 (46%)	**107 (45%)**

#### Specific policy focus within each health system context

However, a more nuanced picture emerged when studying how the specific focus of and specific topics addressed in policy documents are distributed within each health system context (summarised in Table 3). For the United States, two-thirds (*n* = 58, 67%) of documents focused on diagnostic safety, and the remaining third on generic patient safety (*n* = 28; 33%). For example, AHRQ in the United States has produced a series of issue briefs dedicated to diagnostic safety outlining implications and actionable steps to consider for individual clinicians, speciality groups and health care organisations to ensure safety before, during and beyond the immediate diagnostic encounter (10 issue briefs had been published at time of data collection, with a total of 27 published by mid 2025) [24]. A mirror image to the US documents was captured in AUS/AoNZ, with two-thirds (*n* = 102, 68%) of documents focusing on patient safety and one third (*n* = 49, 32%) on diagnostic safety. Many generic safety documents only mentioned diagnostic safety among a list of common patient safety concerns without providing further background information or recommended actions. For example:

The themes of delays (diagnosis, treatment and assessment) and unexpected or adverse outcomes from treatment are common across both events reported to the Commission from the health sector and in consumer complaints made to the Health and Disability Commissioner. 00110 [AUS/AoNZ, Te Tāhū Hauora Health Quality & Safety Commission New Zealand (HQSCNZ) [25]]

When poorly conducted, clinical handover can result in serious adverse events arising from delays in diagnosis or treatment, miscommunication about tests, and the administration of incorrect treatments or medications. 00070 [AUS/AoNZ, Australian Commission on Safety and Quality in Health Care (ACSQHC) [26]]

**Table 3:** Specific focus (generic patient safety vs diagnostic safety) and specific topic category of policy documents within each health system context

	Health systems context			
Specific focus/topic category	United States (n = 86)		AUS/AoNZ (n = 151)	
	Generic PS	Dedicated DxS	Generic PS	Dedicated DxS
**Total, n (%)**	**28 (33%)**	**58 (67%)**	**102 (68%)**	**49 (32%)**
*Clinical process, n (%)*	*2 (7%)*	*7 (12%)*	*12 (12%)*	*7 (14%)*
Adverse events,n	2	5	8	-
Prevention, n	-	1	4	2
Variation, n	-	-	-	5
Other, n	-	1	-	-
*Communication, n (%)*	*4 (14%)*	*3 (5%)*	*18 (18%)*	*1 (2%)*
Complaints, n	-	-	1	-
Disclosure, n	-	-	4	-
Transitions of care, n	3	-	8	-
Other, n	1	3	5	1
*Diagnostic Testing, n (%)*	*3 (11%)*	*1 (2%)*	*10 (10%)*	*2 (4%)*
Imaging, n	3	1	4	1
Pathology, n	-	-	5	-
Other, n	-	-	1	1
*Disease, n (%)*	*0 (0%)*	*1 (2%)*	*22 (22%)*	*22(45%)*
Allergy/Anaphylaxis, n	-	-	2	-
Cancer, n	-	-	2	-
Vascular, n	-	-	2	3
Deterioration, n	-	-	2	1
Infection, n	-	-	5	4
Maternal Health, n	-	1	2	2
Neurology, n	-	-	1	7
Other, n	-	-	3	5
*Education, n (%)*	*2 (7%)*	*3 (5%)*	*0 (0%)*	*0 (0%)*
*Health IT, n (%)*	*0 (0%)*	*2 (3%)*	*1 (1%)*	*0 (0%)*
*Measurement, n (%)*	*0 (0%)*	*6 (10%)*	*0 (0%)*	*0 (0%)*
*Other, n (%)*	*8 (29%)*	*8 (14%)*	*25 (24%)*	*11 (22%)*
*People, n (%)*	*2 (7%)*	*2 (3%)*	*6 (6%)*	*1 (2%)*
CALD, n	1	-	1	-
Children	-	-	3	1
Indigenous, n	-	-	1	-
Nursing, n	-	2	-	-
Older patients, n	-	-	1	-
Veterans, n	1	-	-	-
*Research, n (%)*	*7 (25%)*	*25 (43%)*	*8 (8%)*	*3 (6%)*
Funding	6	21	4	1
Non-specific	1	4	4	2

Both contexts had the greatest proportion of generic patient safety documents classified as *other* (US: *n* = 8, 29%; AUS/AoNZ: *n* = 25, 24%; including annual or patient safety reports, safety goals, priorities etc), yet diverged greatly in relation to remaining highly relevant topics: *disease* and *research*. A reminder that most documents covered various topics; however, we coded each into a single exclusive category, determined by first topic aspect mentioned in the title.

*Disease* was the highest and second highest featured content topic in AUS/AoNZ generic patient safety (*n* = 22, 22%) and diagnostic safety documents (*n* = 22, 45%), respectively, but hardly featured in US documents (*n* = 1, 2%; diagnostic safety only). In relation to *disease*, neurology (e.g. dementia, delirium), infections (e.g. sepsis), and (cardio-)vascular conditions (e.g. stroke) were most often mentioned in diagnostic safety documents. AUS/AoNZ documents focused on disease were largely published by ACSQHC (*n* = 25) and HQSCNZ (*n* = 10) in the form of practical/clinical guidelines (*n* = 15, diagnostic safety *n* = 7) or clinical care standards (*n* = 11; diagnostic safety *n* = 7) describing best practice in the diagnosis and care of certain diseases or conditions (e.g. 00139 “Sepsis Clinical Care Standard” AUS/AoNZ, ACSQHC [27] or 00517 “Guide to improving the use of antibiotics in the management of urinary tract infections in aged residential care” AUS/AoNZ, HQSCNZ [28]. Concerning disease-focused documents, the clinical care standards published by ACSQHC (stroke [29] and comprehensive care standards [30]) show increasing awareness of diagnostic safety and include actionable steps for patients and clinicians, as well as indicators for local monitoring in supporting documents; for example:

Quality statement 1 – Could it be sepsis? […] To improve the recognition and early detection of sepsis in all clinical presentations by using a structured and evidence-based approach to screening and decision-making. 00139 [AUS/AoNZ, ACSQHC [27]]

Avoiding diagnostic error. Diagnostic error is a breakdown in the process of identifying a condition, disease or injury. […] When a patient is not responding to treatment, clinicians should also consider ‘what else could this be?’, particularly when deterioration continues despite treatment. This may require minor or major modifications to the comprehensive care plan. 00001 [AUS/AoNZ, ACSQHC [30]]

In this study, similar disease-focused guidance for diagnostic safety in US policy was only identified in emergency medicine guidelines, though that may soon change with the implementation of diagnostic safety activities via the Council of Medical Specialty Societies [31, 32].

In contrast to the AUS/AoNZ *disease* focus, *research* was highlighted most often in US generic patient safety (*n* = 7, 25%) and diagnostic safety (*n* = 25, 43%) documents, whereas it only had a marginal focus (6–8%) in AUS/AoNZ documents (see Table 3). US documents concerned with diagnostic safety related research were predominantly published by one national health care quality agency (*n* = 13, AHRQ), one national advocacy organisations (*n* = 4; Society to Improve Diagnosis in Medicine (SIDM)), as well as advocacy and philanthropy (*n* = 5, e.g. Gordon and Betty Moore Foundation, The Leapfrog Group). Below an example of research focus from AHRQ:

AHRQ understands that less than 1% of federal health care research spending has been dedicated to diagnostic safety research […] The Committee is concerned about the lack of dedicated research into improving how we diagnose medical conditions, especially given the magnitude of the public health burden of diagnostic failures that lead to patient harm. 00313 [US, AHRQ [33]]

Overall, policy produced by national medical research funding agencies in Australia (NHMRC, Medical Research Future Fund (MRFF)) or Aotearoa New Zealand (Health Research Council of New Zealand (HCRNZ) predominantly focused on improving methods and timeliness of diagnosis overall and for specific diseases (e.g. dementia or cancer) rather than broader research funding aimed at identifying and mitigating diagnostic hazards and pitfalls – a subtle but important difference in emphasis. Examples of disease-focused documents include:

Continue to allocate specific funding to support dementia research on prevention, early diagnosis, quality care and treatments. 00335 [AUS/AoNZ, NHMRC [34]]

Other grants focus on research to prevent illness from occurring in the first place or improving diagnosis. 00251 [AUS/AoNZ, MRFF [35]]

Developing new and better ways to prevent, predict, diagnose and treat mental and physical ill-health and forecast the path, severity and impact of illness. 00339 [AUS/AoNZ, HRCNZ [36]]

Only US documents focused on education (5–7%) and measurement (*n* = 6, 10%, diagnostic safety only), predominantly produced by AHRQ (*n* = 4) and the National Quality Forum (NQF; *n* = 2). Content related to clinical processes, communication, diagnostic testing, and people was distributed roughly equally across generic patient safety and diagnostic safety documents across both contexts (see Table 3). However, while both regions highlighted adverse events within clinical processes equally, AUS/AoNZ documents showed the greater (or sole) focus on prevention and variation.

### Emergency medicine guidelines

Table 4 provides a summary of emergency medicine guidelines across both health system contexts, including their diagnostic focus (e.g. starting with or working towards a diagnosis). A brief note on the format of emergency guidelines: the US clinical guidelines were all published as academic journal articles with lengthy introduction/background sections, references and appendices whereas the AUS/AoNZ clinical guidelines were significantly shorter; with 10 of them contained in a guideline on diagnostic imaging which only provided flowcharts for diagnostic workup with short comments.

**Table 4:** Emergency medicine guidelines across health system contexts, pages, and diagnostic focus

	Health system context		
Guideline characteristics	United States	AUS/AoNZ	Total
Emergency medicine guidelines, n (%)	20 (66%)	16 (44%)	**36 (100%)**
Number of pages, n (%)	662 (89%)	85 (11%)	**747 (100%)**
Work towards diagnosis, n (%)	12 (60%)	9 (56%)	**21 (58%)**
Start with diagnosis, n (%)	2 (10%)	5 (31%)	**7 (19%)**
Dual purpose, n (%)	5 (25%)	0 (0%)	**5 (14%)**
Not diagnosis related, n (%)	1 (5%)	2 (13%)	**3 (8%)**

Overall, more than half of all guidelines in each context assisted clinicians in working towards a diagnosis (*n* = 21, 58%; US: *n* = 12, 60%, AUS/AoNZ: *n* = 9, 56%). The remainder of all guidelines started with a diagnosis (*n* = 7, 19%), had a dual purpose (*n* = 5, 14%) or were not related to diagnosis (*n* = 3, 8%; e.g. procedural guidelines). A quarter of US emergency medicine guidelines, compared to none of the AUS/AoNZ guidelines, had a dual purpose of guiding clinician towards a diagnosis and assisting with management or disposition decisions for patients with established diagnosis.

Across both contexts, most emergency guidelines (US: *n* = 15, 75%, AUS/AoNZ: *n* = 13, 81%) included comments related to ensuring diagnostic safety, e.g. diagnostic accuracy of investigations, threat of delayed/missed diagnoses or (red flag) diagnoses to consider:

CT – high sensitivity (90%) if performed within 24 hours of haemorrhage but a normal CT does not exclude haemorrhage. GC055 [AUS/AoNZ, ACEM [37]]

Improved patient safety by decreasing the risk of missing an ectopic pregnancy among patients with a low b-hCG value. […], the potential for earlier diagnosis of a viable intrauterine pregnancy in many patients will likely reduce the need for further follow-up testing for ectopic pregnancy. GC008 [US, ACEP [38]]

Be aware of possibility of simultaneous intra-uterine pregnancy and ectopic pregnancy. GC050 [AUS/AoNZ, ACEM [39]]

In the evaluation of ED headache, LP after a normal head CT is a long-standing diagnostic regimen that will occasionally reveal alternative diagnoses. If the LP is no longer performed, these diagnoses may be missed, particularly in patients for whom other diagnoses remain in the differential, eg, meningitis. GC010 [US, ACEP [40]]

Overall, nine guidelines featured comments about excluding diagnoses (US: *n* = 6, 30%, AUS/AoNZ: *n* = 3, 19%) with the majority (*n* = 6) related to ruling out vascular events (e.g., myocardial infarction, abdominal aortic aneurysm, subarachnoid haemorrhage, pulmonary embolism). About a fifth of AUS/AoNZ (*n* = 3, 19%) and a quarter of US guidelines (*n* = 5, 25%) did *not* feature diagnostic safety comments.

In relation to featured diseases/symptoms, emergency medicine guidelines focused on vascular events (*n* = 10), followed by accidents (*n* = 6; e.g. blunt head or abdominal trauma), other presentations (*n* = 5, e.g. seizures, mental health), pain (*n* = 3), (symptoms of) infection (*n* = 3), kidney health (*n* = 2) and maternal health (*n* = 2). Four documents provided procedural guidance, e.g. on the use of sedation or pathology testing. There was considerable topical overlap across health system contexts, with seven US guidelines (35%) having an AUS/AoNZ equivalent with a similar or partially similar clinical focus. Named target populations included predominately adults (*n* = 20), adults and children (*n* = 4), children (*n* = 2) and women (*n* = 2). While all US guidelines explicitly identified target populations, only half (*n* = 8, 50%) of AUS/AoNZ guidelines gave similar information.

All twenty (100%) US guidelines explicitly stated that the guideline was based on evidence (e.g. *“This clinical policy is based on a systematic review with critical analysis of the medical literature meeting the inclusion criteria.”* [CG010, ACEP [40]], compared with 75% (*n* = 12) of AUS/AoNZ guidelines (e.g. “*a working group […] developed the following imaging guidelines, using available evidence and best practice”* [CG055, ACEM [37]]. Most US emergency medicine guidelines (*n* = 16, 80%) included a legal disclaimer *“This clinical policy is not intended to represent a legal standard of care for emergency physicians”* (e.g. GC008, ACEP [38]), whereas only two (13%) recently published AUS/AoNZ emergency medicine guidelines did:

*The Australasian College for Emergency Medicine does not accept any legal liability or responsibility for any injury, loss or damage incurred by use of, or reliance on, information provided in this resource*. CG041 [AUS/AoNZ, ACEM [41]]

Medicolegal obligations vary by country, state or territory. Doctors must be aware of relevant legal and institutional requirements and procedures for the jurisdiction and organisation in which they work. CG043[AUS/AoNZ, ACEM [42]]

## Discussion

This study provides the first comparative analysis of how diagnostic safety is reflected and prioritised in national policy documents across the United States and Australia/Aotearoa New Zealand. Across both health system contexts, awareness of diagnostic safety has grown, with half of all diagnosis-focused documents published since 2019, following the NASEM [1] report identifying diagnostic safety as an international patient safety issue.

Diagnostic safety was more prominent in the United States, supported by key government and stakeholder involvement (such as AHRQ and SIDM); whereas AUS/AoNZ documents focused more on generic patient safety - only marginally addressing diagnostic safety and offering limited actionable guidance.

Differences also in the thematic focus of diagnostic safety policy likely reflect broader health system priorities. In AUS/AoNZ, national agencies concentrated on *disease*-specific clinical care standards [43] such as stroke [29], sepsis [27] and acute coronary syndrome [44]; all “Big Three” conditions particularly prone to harmful misdiagnosis [45]. Research funding similarly targeted improving diagnosis for particular diseases [16] rather than health services-based patient safety research [46]. In contrast, US policy documents framed diagnostic safety as a systems issue, building an evidence-base supported by long-called-for dedicated research funding via AHRQ [1, 16, 47, 48]. US publications included novel encouragement to detect and measure diagnostic safety events (e.g. Joint Commission’s Patient Safety Goals [49], NQF Serious Reportable Events 2011 [50], AHRQ supported national common reporting format for diagnostic safety events [51] and more recently NQFs Advancing Measurement of Diagnostic Excellence initiative [52]). Australia and Aotearoa New Zealand currently have no comparable national measures of diagnostic safety.

Emergency medicine guidelines in both contexts clearly focused on diagnostic safety providing actionable advice such as how to exclude red flag diagnoses; and manage vascular conditions among the “Big Three” [45]. Yet, a quarter of guidelines failed to comment on diagnostic safety, leaving room for improvement. Similarly, while over half of guidelines focused on establishing a diagnosis, a quarter emphasised treatment recommendations, presupposing accurate diagnosis in the emergency department. Literature has discussed how the scope of emergency medicine in relation to diagnostic goals might differ between those practising in the field and those outside it (patients, non-emergency medicine clinicians) [53]. According to Schiff et al. [14] “by definition, we expect [emergency medicine] physicians to triage and treat emergencies, not thoroughly work up every problem patients have,” suggesting that emergency medicine prioritises diagnosing life- and limb threatening emergencies over providing patients with definite diagnoses, which may explain the high rates of symptom-based discharge diagnosis [54]. Legal disclaimers were universal in US emergency medicine guidelines but less frequent in AUS/AoNZ, reflecting differing medico-legal contexts and concerns about diagnostic safety related malpractice claims [55, 56], with Aotearoa New Zealand’s no-fault compensation scheme fostering a less litigious culture [57].

### Implications for policy and practice

The absence of a centralised patient safety research agency comparable to AHRQ means AUS/AoNZ works from a distributed model of safety and quality spread across multiple government agencies and regulatory bodies, limiting sustained federal-level funding for diagnostic safety. Many US diagnostic safety documents focused on research funding, and in comparison to AUS/AoNZ, the United States benefit from dedicated policy briefs [24], federal funding [33, 58–60], national diagnostic safety measures [49–52] and a growing community [11, 47, 61, 62]. Nevertheless, US funding remains vulnerable to political [63, 64], budgetary and philanthropic fluctuations [58, 65], risking potentially damaging and irreversible changes [66, 67]. Notably, AHRQs annual budget, responsible for healthcare quality and safety research, remains under one percent of the disease-focused National Institutes of Health (NIH) research budget for, and both were severely reduced in 2025 [68, 69].

Although national diagnostic safety policies and emergency guidelines exist across US and AUS/AoNZ contexts, recommendations were often non-binding and coordinated national implementation by organisations or clinicians not guaranteed [12, 70, 71]. Financial incentives, mandates or healthcare accreditation-linked measures have been proposed to improve uptake but each has limitations [12, 71–74]. In Australia, ACSQHCs National Safety and Quality Health Service Standards are tied to accreditation; but disease-focused clinical care standards are not mandated. Similarly, in the United States, AHRQs diagnostic safety briefs and recommended practices are not binding. Australia’s Independent Health and Aged Care Pricing Authority enforces quality-based funding penalties for sentinel events [75], hospital acquired complications and avoidable hospital readmissions [76, 77], which include diagnostic safety relevant items, e.g. blood stream infections [77] However, recent declines in complications and readmissions cannot yet be directly attributed to these newly introduced safety measures [76].

### Strengths and limitations

Our study’s strength lies in providing the first systematic comparative analysis of how diagnostic safety is currently integrated in national healthcare and research policy documents and emergency medicine guidelines. Limitations include the focus on national-level documents (which may not capture state or institutional variation), use of single topic coding (which may have shaped impressions of particular countries not discussing certain aspects), and a reliance on keyword-guided searches (which may have missed content embedded in broader safety networks) and web-hosted policy sources (which may have underrepresented earlier or superseded policies). Additionally, the variability in purpose and scope of emergency medicine guidelines produced by specialist colleges may have affected comparability across health contexts. For example, the AUS/AoNZ college might prioritise training, education and professional standards [78] over detailed, evidence-based clinical guidance [79], while still contributing expert advice to broader patient safety initiatives or external resource repositories such as “GOODEM – Guidelines Of Others Displayed-Emergency Medicine” [80]. Consequently, US guidelines were lengthy academic reviews, while AUS/AoNZ guidelines were briefer and more practically oriented.

Future qualitative research could examine international perspectives of policy makers, clinicians and consumers involved in creating and implementing diagnostic safety policies to explore how national priorities are developed and translated into practice. More detailed analysis of multiple specific topic categories present in policy documents could inform strategic approaches for each relevant policy focus to be more effectively addressed in practice.

## Conclusion

This study analysed policy documents and emergency medicine guidelines from US and AUS/AoNZ contexts to trace the development and focus of such documents in relation to diagnostic safety – a field that has so far received limited attention in the health policy space. We identified a growing international awareness for diagnostic safety- with the United States currently producing more prevalent and dedicated diagnostic safety policy documents compared to AUS/AoNZ. Diagnostic safety comments featured equally in US and AUS/AoNZ emergency medicine guidelines. Comparing diagnostic safety policies across health system contexts revealed diverse approaches and priorities. This new knowledge can help identify strengths and shortcomings within a particular country as well as related barriers or facilitators within health systems and research landscapes and thus inform targeted systemic change and diagnostic safety policy development in the future. Following on from our findings, US policy makers should leverage existing disease-specific guidelines and focus on practical implementation strategies in addition to research funding. Australian policymakers should support the ACSQHC to coordinate diagnostic safety initiatives [26, 43, 81, 82] with increased dedicated research funding. Both Australia and Aotearoa New Zealand should prioritise investing in the development of a comprehensive framework for implementing diagnostic safety measures across healthcare settings.

These findings underscore the need for a balanced approach incorporating both research investment and practical implementation strategies to improve diagnostic safety and patient outcomes nationally, and in time, globally.

## Data Availability

The dataset developed and/or analysed for the current study is available from the corresponding author on reasonable request.

## Abbreviations

ACEM: Australasian College for Emergency Medicine (Australia/Aotearoa New Zealand)
ACEP: American College of Emergency Physicians (United States)
ACSQHC: Australian Commission on Safety and Quality in Health Care (Australia)
AHRQ: Agency for Healthcare Research and Quality (United States)
AoNZ: Aotearoa New Zeland
CMS: Centers of Medicare and Medicaid Services (United States)
DHHS: Department of Health and Human Services (United States)
GBMF: The Gordon and Betty Moore Foundation (United States)
HQSCNZ: Te Tāhū Hauora Health Quality & Safety Commission (Aotearoa New Zealand)
HRCNZ: Health Research Council of New Zealand (Aotearoa New Zealand)
MRFF: Medical Research Future Fund (Australia)
NASEM: National Academies of Sciences, Engineering, and Medicine (United States)
NHMRC: National Health and Medical Research Council (Australia)
NQF: National Quality Forum (United States)
SIDM: Society to Improve Diagnosis in Medicine (United States)

## References

[1] National Academies of Science Engineering & Medicine (NASEM). Improving diagnosis in health care. Washington DC: NASEM; 2015.

[2] Graber ML. The incidence of diagnostic error in medicine. BMJ Qual Saf. 2013;22(Suppl 2):ii21–7. 10.1136/bmjqs-2012-001615 23771902 10.1136/bmjqs-2012-001615 PMC3786666

[3] Newman-Toker DE, Peterson SM, Badihian S, Hassoon A, Nassery N, Parizadeh D, et al. Diagnostic errors in the emergency department: a systematic review. Rockville (MD): Agency for Healthcare Research and Quality (US); 2022. 36574484

[4] Newman-Toker DE, Nassery N, Schaffer AC, Yu-Moe CW, Clemens GD, Wang Z, et al. Burden of serious harms from diagnostic error in the USA. BMJ Qual Saf. 2024;33(2):109. 10.1136/bmjqs-2021-014130 37460118 10.1136/bmjqs-2021-014130 PMC10792094

[5] Croskerry P. The rational diagnostician and achieving diagnostic excellence. JAMA. 2022;327(4):317–18. 10.1001/jama.2021.24988 34994774 10.1001/jama.2021.24988

[6] Dahm MR, Crock C. Understanding and communicating uncertainty in achieving diagnostic excellence. JAMA. 2022;327(12):1127–28. 10.1001/jama.2022.2141 35238876 10.1001/jama.2022.2141

[7] Olson A, Rencic J, Cosby K, Rusz D, Papa F, Croskerry P, et al. Competencies for improving diagnosis: an interprofessional framework for education and training in health care. Diagnosis. 2019;6(4):335–41. 10.1515/dx-2018-0107 31271549 10.1515/dx-2018-0107

[8] Burstin H, Cosby K. Measuring performance of the diagnostic process. JAMA. 2022;328(2):143–44. 10.1001/jama.2022.10166 35737397 10.1001/jama.2022.10166

[9] Runciman WB. Lessons from the Australian patient safety foundation: setting up a national patient safety surveillance system–is this the right model? BMJ Qual Saf. 2002;11(3):246–51. 10.1136/qhc.11.3.246 10.1136/qhc.11.3.246 PMC1743620 12486989

[10] The patient safety and quality improvement act of 2005. Pub. L. 109–41, . part C: U.S.C.; 2005 42 ch 6A subch. VII.

[11] Wachter RM. Diagnostic errors: central to patient safety, yet still in the periphery of safety’s radar screen. Diagnosis. 2014;1(1):19–21. 10.1515/dx-2013-0035 29539982 10.1515/dx-2013-0035

[12] Scott IA, Crock C. An organisational approach to improving diagnostic safety. Aust Health Rev. 2023;47(3):261–67. 10.1071/AH22287 36966762 10.1071/AH22287

[13] Berenson RA, Upadhyay DK, Kaye DR. Placing diagnosis errors on the policy agenda. Princeton, NJ: Robert Wood Johnson Foundation; 2014.

[14] Schiff GD, Kim S, Abrams R, Cosby K, Lambert B, Elstein AS, et al. Diagnosing diagnosis errors: lessons from a multi-institutional collaborative project. In: Henriksen K, Battles J, Marks E, Lewin D, editors. Advances in patient safety: from research to implementation, vol 2, concepts and methodology. Rockville, MD: AHRQ; 2005. p. 255–78. 21249820

[15] Graber ML, Trowbridge R, Myers JS, Umscheid CA, Strull W, Kanter MH. The next organizational challenge: finding and addressing diagnostic error. Jt Comm J Qual Patient Saf. 2014;40(3):102–10. 10.1016/s1553-7250(14)40013-8 24730205 10.1016/s1553-7250(14)40013-8

[16] Zwaan L, El-Kareh R, Meyer AND, Hooftman J, Singh H. Advancing diagnostic safety research: results of a systematic research priority setting exercise. J Gener Intern Med. 2021;36(10):2943–51. 10.1007/s11606-020-06428-3 10.1007/s11606-020-06428-3 PMC8481519 33564945

[17] Australian Medical Council (AMC). Assessment and accreditation of specialist medical programs. 2024 [cited 2025 June]. Available from: https://www.amc.org.au/accredited-organisations/assessment-and-accreditation-of-specialist-medical-programs/.

[18] Australian Medical Council (AMC). Assessment and accreditation of primary medical programs (medical schools). 2024 [cited 2025 June]. Available from: https://www.amc.org.au/accredited-organisations/medical-schools/assessment-and-accreditation-of-primary-medical-programs-medical-schools/.

[19] Dahm MR, Chien LJ, Morris J, Lutze L, Scanlan S, Crock C. Addressing diagnostic uncertainty and excellence in emergency care—from multicountry policy analysis to communication practice in Australian emergency departments: a multimethod study protocol. BMJ Open. 2024;14:e085335. 10.1136/bmjopen-2024-085335 39277199 10.1136/bmjopen-2024-085335 PMC11404230

[20] Dalglish SL, Khalid H, McMahon SA. Document analysis in health policy research: the read approach. Health Policy and Planning. 2020;35(10):1424–31. 10.1093/heapol/czaa064 10.1093/heapol/czaa064 PMC7886435 33175972

[21] Bowen GA. Document analysis as a qualitative research method. Qualitative Res J. 2009;9(2):27–40. 10.3316/QRJ0902027

[22] American College of Emergency Physicians (ACEP). Clinical Policies. 2023. Available from: https://www.acep.org/patient-care/clinical-policies.

[23] Australasian College for Emergency Medicine (ACEM). Standards and Advocacy Library. 2023. Available from: https://acem.org.au/Search-Pages/Standards-and-Advocacy-Library.

[24] Agency for Healthcare Research and Quality. (AHRQ). AHRQ papers on diagnostic safety topics - issue briefs 2024. Available from: https://web.archive.org/web/20240809034050/https://www.ahrq.gov/diagnostic-safety/resources/issue-briefs.html.

[25] Te Tāhū Hauora health quality & safety commission New Zealand (HQSCNZ). A window on the quality of New Zealand’s health care 2018 [January 2024]. Available from: https://web.archive.org/web/20240809035056/https://www.hqsc.govt.nz/assets/Our-data/Publications-resources/Window-Jun-2018.pdf.

[26] Australian Commission for Safety and Quality in Health Care (ACSQHC). Creating safer. Better health Care - the impact of the national safety and quality health service standards. 2018. Available from: https://web.archive.org/web/20240809033348/https://www.safetyandquality.gov.au/publications-and-resources/resource-library/creating-safer-better-health-care-impact-national-safety-and-quality-health-service-standards.

[27] Australian Commission for Safety and Quality in Health Care (ACSQHC). Sepsis Clinical Care Standard (2022). 2022. Available from: https://web.archive.org/web/20240809033928/https://www.safetyandquality.gov.au/publications-and-resources/resource-library/sepsis-clinical-care-standard-2022.

[28] Te Tāhū Hauora Health Quality & Safety Commission New Zealand (HQSCNZ). Guide to improving the use of antibiotics in the management of urinary tract infections in aged residential care 2022. Available from: https://web.archive.org/web/20240809035447/https://www.hqsc.govt.nz/resources/resource-library/guide-to-improving-the-use-of-antibiotics-in-the-management-of-urinary-tract-infections-in-aged-residential-care/.

[29] Australian Commission for Safety and Quality in Health Care (ACSQHC). Acute Stroke Clinical Care Standard (2019). 2019. Available from: https://web.archive.org/web/20240809033257/https://www.safetyandquality.gov.au/publications-and-resources/resource-library/acute-stroke-clinical-care-standard-2019.

[30] Australian Commission for Safety and Quality in Health Care (ACSQHC). Implementing the comprehensive care standard - clinical assessment and diagnosis 2020. Available from: https://web.archive.org/web/20240809033529/https://www.safetyandquality.gov.au/publications-and-resources/resource-library/implementing-comprehensive-care-standard-clinical-assessment-and-diagnosis.

[31] Council of Medical Specialty Societies. Promoting Diagnostic Excellence Across Medicine. 2023. Available from: https://web.archive.org/web/20240809034120/https://cmss.org/promoting-diagnostic-excellence/.

[32] Council of Medical Specialty Societies. Diagnostic Feedback in Clinical Registries. 2023. Available from: https://web.archive.org/web/20240809034229/https://cmss.org/diagnostic-feedback-in-clinical-registries/.

[33] Department of Health and Human Services (DHHS). Fy 2019 congressional justification—budget estimates for appropriations committees - national institute for research on safety and quality (NIRSQ). 2018. Available from: https://web.archive.org/web/20230527131002/https://www.ahrq.gov/sites/default/files/wysiwyg/cpi/about/mission/budget/2019/NIRSQ.pdf.

[34] National health and medical research council (NHMRC). Health priorities 2021–2024. n.d. Available from: https://web.archive.org/web/20240809040141/https://www.nhmrc.gov.au/research-policy/research-priorities/nhmrc-health-priorities.

[35] Medical research future fund (MRFF). Budget 2022–23: funding for medical research 2022. Available from: https://www.health.gov.au/sites/default/files/documents/2022/03/budget-2022-23-funding-for-medical-research.pdf.

[36] Health research council of New Zealand (HRCNZ). The New Zealand health research Prioritisation framework. 2019. Available from: https://web.archive.org/web/20240809035710/https://www.hrc.govt.nz/resources/new-zealand-health-research-prioritisation-framework.

[37] Australasian College for Emergency Medicine (ACEM). Guidelines on diagnostic imaging - investigation of a possible subarachnoid haemorrhage 2012. Available from: https://web.archive.org/web/20240329130004/https://acem.org.au/getmedia/b7f67701-7e80-4e06-b87e-9193310415b0/Guidelines_on_Diagnostic_Imaging.

[38] American College of Emergency Physicians (ACEP). Early Pregnancy. 2018. Available from: https://web.archive.org/web/20240809032348/https://www.acep.org/patient-care/clinical-policies/early-pregnancy/.

[39] Australasian College for Emergency Medicine (ACEM). Guidelines on diagnostic imaging - first trimester pregnancy – pain or bleeding 2012. Available from: https://web.archive.org/web/20240329130004/https://acem.org.au/getmedia/b7f67701-7e80-4e06-b87e-9193310415b0/Guidelines_on_Diagnostic_Imaging.

[40] American College of Emergency Physicians (ACEP). Headache. 2019. Available from: https://web.archive.org/web/20240809032806/https://www.acep.org/patient-care/clinical-policies/headache/.

[41] Australasian College for Emergency Medicine (ACEM). Guidelines on management of respiratory disease outbreaks 2020. Available from: https://web.archive.org/web/20240809031555/https://acem.org.au/getmedia/a5d70088-7ed8-4a23-aa0c-a06f1082be5c/Management-of-Respiratory-Disease-Outbreaks.

[42] Australasian College for Emergency Medicine (ACEM). Guideline on surgery or intervention for patients at end-of-life 2022. Available from: https://web.archive.org/web/20240809031720/https://acem.org.au/getmedia/35e5d054-980e-40cf-a0f2-da9d6cf416ff/PG67(G)-End-of-life-Care.

[43] Australian Commission for Safety and Quality in Health Care (ACSQHC). Clinical care standards 2024. Available from: https://web.archive.org/web/20240809033953/https://www.safetyandquality.gov.au/standards/clinical-care-standards.

[44] Australian Commission for Safety and Quality in Health Care (ACSQHC). Acute Coronary Syndromes Clinical Care Standard (2019). 2019. Available from: https://web.archive.org/web/20240809033456/https://www.safetyandquality.gov.au/publications-and-resources/resource-library/acute-coronary-syndromes-clinical-care-standard-2019.

[45] Newman-Toker DE, Schaffer AC, Yu-Moe CW, Nassery N, Tehrani ASS, Clemens GD, et al. Serious misdiagnosis-related harms in malpractice claims: the “big three” – vascular events, infections, and cancers. Diagnosis. 2019;6(3):227–40. 10.1515/dx-2019-0019 31535832 10.1515/dx-2019-0019

[46] Kingsley T. Advocating for health services research and AHRQ. SGIM Forum. 2018;41(10):13–14.

[47] Henriksen K, Dymek C, Harrison MI, Brady PJ, Arnold SB. Challenges and opportunities from the agency for healthcare research and quality (AHRQ) research summit on improving diagnosis: a proceedings review. Diagnosis. 2017;4(2):57–66. 10.1515/dx-2017-0016 29536924 10.1515/dx-2017-0016

[48] Graber ML. Diagnostic errors in medicine: a case of neglect. Jt Comm J Qual Patient Saf. 2005;31(2):106–13. 10.1016/s1553-7250(05)31015-4 15791770 10.1016/s1553-7250(05)31015-4

[49] The Joint Commission. National patient safety Goals® effective January 2022 for the hospital program 2022. Available from: https://web.archive.org/web/20240809040504/https://www.jointcommission.org/-/media/tjc/documents/standards/national-patient-safety-goals/2022/npsg_chapter_hap_jan2022.pdf.

[50] National Quality Forum (NQF). Serious reportable events. 2011. Available from: https://web.archive.org/web/20240809040213/https://www.qualityforum.org/Topics/SREs/List_of_SREs.aspx.

[51] Patient Safety Organizations Privacy Protection Center. Common formats for event reporting - diagnostic safety version 1.0 2023. Available from: https://web.archive.org/web/20240809040327/https://www.psoppc.org/psoppc_web/publicpages/commonFormatsDSV1.0.

[52] Williams-Bader J, Km M, Drye EE. Advancing measurement of diagnostic excellence for better healthcare. Joint Comm J Qual Patient Saf. 2025;51(5):386–87. 10.1016/j.jcjq.2025.01.011 40057431 10.1016/j.jcjq.2025.01.011

[53] Than MP, Flaws DF. Communicating diagnostic uncertainties to patients: the problems of explaining unclear diagnosis and risk. BMJ Evidence-Based Med. 2009;14(3):66–67. 10.1136/ebm.14.3.66 10.1136/ebm.14.3.66 19483015

[54] Wen LS, Espinola JA, Kosowsky JM, Ca C. Do emergency department patients receive a pathological diagnosis? A nationally-representative sample. West J Emerg Med. 2015;16(1):50–54. 10.5811/westjem.2014.12.23474 25671008 10.5811/westjem.2014.12.23474 PMC4307726

[55] Kessler DP, Summerton N, Graham JR. Effects of the medical liability system in Australia, the UK and the USA. Lancet. 2006;368(9531):240–46. 10.1016/S0140-6736(06)69045-4 16844494 10.1016/S0140-6736(06)69045-4

[56] Bird S. Diagnostic tests and litigation. Aust Prescr. 2012;35:106–07. 10.18773/austprescr.2012.045

[57] Wallis KA. No-fault, no difference: no-fault compensation for medical injury and healthcare ethics and practice. Br J Gen Pract. 2017;67(654):38–39. 10.3399/bjgp17X688777 28034949 10.3399/bjgp17X688777 PMC5198606

[58] Department of Health and Human Services (DHHS). Fiscal Year 2022 agency for healthcare research and quality justification of estimates for appropriations committees 2021. Available from: https://web.archive.org/web/20240809034401/https://www.ahrq.gov/cpi/about/mission/budget/2022/index.html.

[59] Department of Health and Human Services (DHHS). 2022 AHRQ justification of estimates for appropriations committees 2021. Available from: https://web.archive.org/web/20240809034555/https://www.ahrq.gov/sites/default/files/wysiwyg/cpi/about/mission/budget/2022/FY2022_CJ.pdf.

[60] Agency for Healthcare Research and Quality (AHRQ). Special emphasis notice (SEN). AHRQ announces interest in research on diagnostic errors in ambulatory care settings. 2007. Available from: https://web.archive.org/web/20240809033807/https://grants.nih.gov/grants/guide/notice-files/not-hs-08-002.html.

[61] Satterfield K, Rubin JC, Friedman CP. Toward a learning ecosystem for diagnostic excellence - a white paper prepared for the Gordon and Betty Moore Foundation: University of Michigan. 2018 [January 2024]. Available from: https://web.archive.org/web/20240809040430/https://deepblue.lib.umich.edu/handle/2027.42/145487.

[62] Community improving diagnosis in medicine (CIDM). Community improving diagnosis in medicine (CIDM). 2025 [cited 2025 June]. Available from: https://www.improve-dx.org/.

[63] The White House. H.R. 5894 — Departments of labor, health and human services, and education, and related agencies appropriations act, (November 13, 2023) 2023. Available from: https://web.archive.org/web/20240809041631/https://www.whitehouse.gov/wp-content/uploads/2023/11/H.R.-5894-%E2%80%94-Departments-of-Labor-Health-and-Human-Services-and-Education-and-Related-Agencies-Appropriations-Act-2024-SAP-Final.pdf.

[64] Vogel L. Trump administration shutters clinical guidelines database. Cmaj. 2018;190(27):E841. 10.1503/cmaj.109-5624 29986863 10.1503/cmaj.109-5624 PMC6041247

[65] Gordon and Betty Moore Foundation (GBMF). Moore foundation concludes patient care program and establishes center for diagnostic excellence at UCSF 2023. Available from: https://web.archive.org/web/20240809034832/https://www.moore.org/article-detail?newsUrlName=moore-foundation-concludes-patient-care-program-and-establishes-center-for-diagnostic-excellence-at-ucsf.

[66] Allen A. What’s lost: trump whacks tiny agency that works to make the Nation’s health care safer. 2025 [cited 2025 June]. Available from: https://kffhealthnews.org/news/article/patient-safety-health-agency-dissolved-doge-federal-workforce-cuts-ahrq/.

[67] Wu AW, Toker DN, Thomas EJ, Buckle P, Sibal A, Letaief M, et al. Preserving AHRQ patient safety network (PSNet): an essential tool for patient safety practitioners. J Patient Saf And Risk Manag. 2025;30(2):59–61. 10.1177/25160435251339942

[68] Neergaard L, Casey M. Judge extends temporary block to huge cuts in national institutes of health research funding. Ap News. 2025 [cited 2025 June]. Available from: https://apnews.com/article/trump-nih-medical-research-funding-cut-indirect-costs-a75b8d7d56a29f1e880859d79ef744e4.

[69] Rosenthal E. How trump aims to slash federal support for research. Public health, and Medicaid KFF health News. 2025. Available from: https://kffhealthnews.org/news/article/health-care-spending-cuts-research-trump-administration-tariffs-public-health/.

[70] Mercuri M, Sherbino J, Sedran RJ, Frank JR, Gafni A, Norman G. When guidelines don’t guide: the effect of patient context on management decisions based on clinical practice guidelines. Academic Med. 2015;90(2):191–96. 10.1097/acm.0000000000000542 10.1097/ACM.0000000000000542 25354075

[71] Khan S, Cholankeril R, Sloane J, Offner A, Bradford A, Matin R, et al. Current state of diagnostic safety: implications for research, practice, and Policy: AHRQ (Agency for Healthcare Research and Quality). 2024. Available from: https://web.archive.org/web/20240809035859/https://www.ahrq.gov/diagnostic-safety/resources/issue-briefs/dxsafety-current-state.html,.

[72] Berenson RA, Singh H. Payment innovations to improve diagnostic accuracy and reduce diagnostic error. Health Aff (Millwood). 2018;37(11):1828–35. 10.1377/hlthaff.2018.0714 30395510 10.1377/hlthaff.2018.0714

[73] Duckett S, Jorm C, Moran G, Parsonage H. Safer care saves money: how to improve patient care and save public money at the same time. 2018. Available from: https://web.archive.org/web/20240809034635/https://grattan.edu.au/report/safer-care-saves-money/: Grattan Institute.

[74] Greenfield D, Hinchcliff R, Banks M, Mumford V, Hogden A, Debono D, et al. Analysing ‘big picture’ policy reform mechanisms: the Australian health service safety and quality accreditation scheme. Health Expectations. 2015;18(6):3110–22. 10.1136/bmjopen-2017-020235 25367049 10.1111/hex.12300 PMC5810648

[75] Australian Commission for Safety and Quality in Health Care (ACSQHC). Australian sentinel events list (version 2) specifications 2020. Available from: https://web.archive.org/web/20240809033509/https://www.safetyandquality.gov.au/publications-and-resources/resource-library/australian-sentinel-events-list-version-2-specifications.

[76] Webster SBG, Neville SE, Nobbs J, Ching J, van Gool K. Incorporating safety and quality measures into Australia’s activity-based funding of public hospital services. Health Serv Insights. 2023;16:1–8. 10.1177/11786329231187891 10.1177/11786329231187891 PMC10387671 37529090

[77] Independent Health and Aged Care Pricing Authority (IHACPA). Safety and quality 2024. Available from: https://web.archive.org/web/20240809035748/https://www.ihacpa.gov.au/health-care/pricing/safety-and-quality.

[78] Australasian College for Emergency Medicine (ACEM). About us - role. n.d. Available from: https://web.archive.org/web/20240809032234/https://acem.org.au/Content-Sources/About/About-us.

[79] American College of Emergency Physicians (ACEP). About ACEP. 2024. Available from: https://web.archive.org/web/20240809033311/https://www.acep.org/who-we-are/about-us.

[80] Peripheral Hospitals Emergency Medicine Conference. GOODEM – guidelines of others displayed-emergency medicine 2024. Available from: https://www.phemc.org/guidelines/.

[81] Australian Commission for Safety and Quality in Health Care (ACSQHC). The NSQHS standards 2022. Available from: https://web.archive.org/web/20240809033715/https://www.safetyandquality.gov.au/standards/nsqhs-standards.

[82] Australian Commission for Safety and Quality in Health Care (ACSQHC). National safety and quality medical imaging standards consultation 2024. Available from: https://web.archive.org/web/20240809033658/https://www.safetyandquality.gov.au/favicon.ico.

